# The Mississippi River records glacial-isostatic deformation of North America

**DOI:** 10.1126/sciadv.aav2366

**Published:** 2019-01-30

**Authors:** Andrew D. Wickert, Robert S. Anderson, Jerry X. Mitrovica, Shawn Naylor, Eric C. Carson

**Affiliations:** 1 Department of Earth Sciences and Saint Anthony Falls Laboratory, University of Minnesota, 116 Church St. SE, Minneapolis, MN 55455, USA.; 2 Institute of Arctic and Alpine Research and Department of Geological Sciences, University of Colorado, 4001 Discovery Dr., Boulder, CO 80303, USA.; 3 Department of Earth and Planetary Sciences, Harvard University, 20 Oxford St., Cambridge, MA 02138, USA.; 4 Center for Geospatial Data Analysis and Indiana Geological Survey, Indiana University, 611 N. Walnut Grove St., Bloomington, IN 47405, USA.; 5 Wisconsin Geological and Natural History Survey, 3817 Mineral Point Rd., Madison, WI 53705, USA.

## Abstract

The imprint of glacial isostatic adjustment has long been recognized in shoreline elevations of oceans and proglacial lakes, but to date, its signature has not been identified in river long profiles. Here, we reveal that the buried bedrock valley floor of the upper Mississippi River exhibits a 110-m-deep, 300-km-long overdeepening that we interpret to be a partial cast of the Laurentide Ice Sheet forebulge, the ring of flexurally raised lithosphere surrounding the ice sheet. Incision through this forebulge occurred during a single glacial cycle at some time between 2.5 and 0.8 million years before present, when ice-sheet advance forced former St. Lawrence River tributaries in Minnesota and Wisconsin to flow southward. This integrated for the first time the modern Mississippi River, permanently changing continental-scale hydrology and carving a bedrock valley through the migrating forebulge with sediment-poor water. The shape of the inferred forebulge is consistent with an ice sheet ~1 km thick near its margins, similar to the Laurentide Ice Sheet at the Last Glacial Maximum, and provides evidence of the impact of geodynamic processes on geomorphology even in the midst of a stable craton.

## Teaser

A deep gash through bedrock now buried beneath Mississippi River sediments formed when massive glaciers warped Earth’s crust.

## INTRODUCTION

The upper Mississippi valley is a 250-m-deep trench incised through the bedrock of the low-relief Paleozoic Plateau of the north-central United States, in the middle of the stable North American craton. Recent work traces its origin to advancing continental ice sheets (*1*), which dammed northeast-flowing rivers into lakes that then overtopped bedrock drainage divides and incised to form the modern integrated Mississippi. Today, the bedrock valley floor of the upper Mississippi River is buried beneath up to 110 m of alluvium, hiding its shape and therefore the topographic evidence of the events that formed it.

**Fig. 1:**
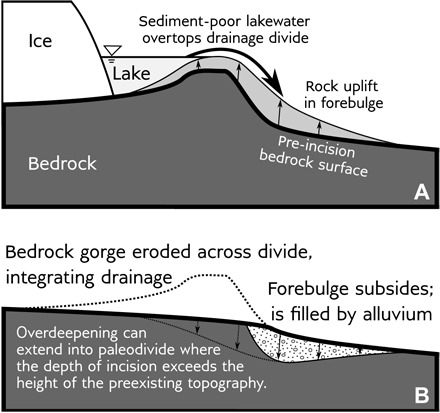
Schematic of drainage integration and forebulge incision. (**A**) An ice sheet dams rivers on one side of a divide, ponding their waters into lakes that overtop the drainage divide. This sediment-poor proglacial lake outflow incises into the paleodivide and the glacial-isostatically uplifting forebulge. (**B**) When the ice retreats, glaciofluvial erosion has cut a gorge across the divide, permanently integrating drainage. The eroded forebulge subsides and aggrades, leaving a sediment-filled cast of part or all of its shape.

Ice-sheet advance and retreat is accompanied by extensive glacial isostatic adjustment (GIA) (*2*), as documented by deformed lake shorelines (*3*, *4*) and in sea-level histories along the coast (*5*). The pattern of GIA-induced vertical displacement includes deep subsidence beneath the ice-sheet footprint, as well as a flexural upwarp, called a “forebulge” or “peripheral bulge,” that forms outboard of the ice margin (*6*). In cratonal continental interiors, subtle GIA-induced changes in surface slope are similar to river gradients (~10^−4^). Such a change may reroute the course of a river (*7*–*9*) or, in the case that we propose here, cause it to incise across the uplifting forebulge. Following this incision, subsidence of the forebulge during and after ice-sheet retreat can produce an overdeepening in the bedrock valley floor that fills with sediment, recording the signature of GIA in the river’s bedrock long profile (Fig. 1).

**Fig. 2:**
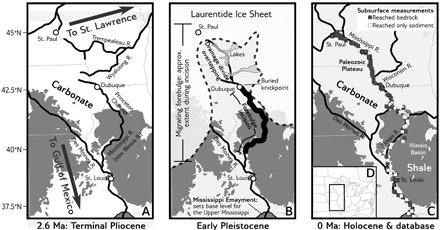
Pleistocene drainage evolution in the upper Mississippi drainage basin. (**A**) The pre-Pleistocene continental divide extended from southern Wisconsin to southern Minnesota (*30*) and had been stable throughout the Cenozoic (*101*). The pre-LGM Mississippi River followed the course of the Illinois River over erodible shales; LGM ice advance pushed it into its current course (*13*). (**B**) Ice advance (drawn schematically; exact pattern unknown) dammed and rerouted former tributaries to the St. Lawrence, causing them to overtop and incise through the paleodivide (*1*). (**C**) Modern rivers and depth to bedrock data points. (**D**) Location map. In map (A to C) backgrounds, shading indicates shale (easily erodible), and carbonate bedrock underlies most of the paleodivide region (fig. S4).

The Mississippi River is the ideal place to seek such a record of GIA. It flows away from the margin of the Laurentide Ice Sheet (LIS) (Fig. 2), which at the Last Glacial Maximum (LGM) was larger than the present-day Antarctic Ice Sheet (*10*). The Mississippi crosses the zone where models of GIA since the LGM predict the presence of an extensive forebulge (*11*) and GPS measurements record ongoing subsidence (“forebulge collapse”) (fig. S1) (*6*). Furthermore, early mapping of bedrock topography (*12*) indicates that the pre-LGM course of the upper Mississippi, the buried Princeton-Illinois bedrock valley (*13*), has a modern slope of less than 3 × 10^−5^, whereas the alluvial bed slope of the modern Mississippi River is 1 × 10^−4^ along the same latitudinal band. This led to speculation—over 50 years ago—that surface deformation associated with the LIS forebulge back-tilted the incised bedrock floor of the ancient Mississippi (*14*). In this study, we assemble a new and comprehensive depth-to-bedrock dataset and use this alongside modeling of GIA and the river long profile to (i) confirm the evidence for a glacial forebulge, (ii) estimate the thickness of the ice sheet that formed the forebulge, and (iii) suggest that the Mississippi must have incised during the earliest significant ice advance across Wisconsin, in the north-central United States, which is constrained (*15*–*18*) to have occurred between 0.8 and 2.5 million years (Ma) before present (B.P.).

## RESULTS

### Mapping the Mississippi long profile

**Fig. 3:**
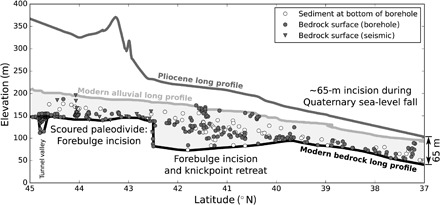
Upper Mississippi long-profile evolution. The Pliocene long profile was reconstructed by identifying ancient river courses and buried strath terraces in statewide depth-to-bedrock data and gridding these sparse elevation markers into a hydrologically consistent DEM (*20*). The modern mainstem alluvial surface lies above the bedrock long profile of the upper Mississippi, which includes the now-abandoned Princeton Channel–Illinois River course. The bedrock long profile may be split into three zones, as indicated on the figure. Downstream of the forebulge-induced overdeepening, a uniform incision signal dominates. Above the forebulge-induced overdeepening, buried bedrock elevations frequently align with the downstream bedrock long profile, indicating that forebulge incision created a narrow inner gorge that lies within a longer-occupied valley floor.

To test the hypothesis that the Mississippi River incised through an early LIS forebulge, we compiled a set of subsurface geotechnical boring and well log data to document the shape of the bedrock floor of the upper Mississippi valley, including former courses of the upper Mississippi River, and supplemented these data with our own passive seismic measurements [following (*19*)]. All data are available in data file S1, are summarized in Figs. 2C and 3, and are described in more detail in the Supplementary Materials and Methods.

In addition to mapping the buried bedrock topography, we built two Mississippi reference surfaces: the late Pliocene bedrock long profile and the modern alluvial long profile (Fig. 3). We reconstructed the Pliocene surface by (i) compiling and rasterizing measurements of the elevation of the bedrock surface, (ii) plotting preglacial river courses based on geologic data and the buried strath terraces that our bedrock topographic compilation revealed, and (iii) reconstructing preglacial (i.e., late Pliocene) topography by building a hydrologically consistent (*20*) digital elevation model (DEM) from these surfaces and river courses. We constructed the modern upper Mississippi long profile by routing flow down a DEM surface and then processing the result to remove the stair-step effect of the locks and dams on the Mississippi River. (See Materials and Methods and the Supplementary Materials for details of both workflows).

The modern bedrock long profile of the Mississippi River (Fig. 3) does not display the idealized concave-up profile of a steady-state river (*21*), and this bears witness to its complex history of glaciation and assembly. Drilling logs and passive seismic surveys reveal a knickpoint at 42.5°N, where the bedrock valley floor drops 50 m over <4 km; it is possible that this drop is more sudden, but the horizontal distance reported here is limited by the resolution of our subsurface data. Downstream of this knickpoint is a 300-km-long reach of anomalously deep flat-to-reverse sloping channel that extends along the pre-LGM Mississippi River course (*13*, *14*) from 42.5°N to 39.7°N (Fig. 2, A and B). Downstream of this overdeepened profile, the bedrock valley floor grades smoothly downward toward the Mississippi Embayment, ~60 m below the modern long profile.

### Glacial isostatic adjustment

To gain insight into the shape and dynamics of the LIS forebulge in the upper Mississippi, we modeled GIA over the only period for which we have good constraints: the LGM to present. We used the ICE-6G ice-sheet reconstruction and the associated one-dimensional mantle viscosity profile VM5a (*11*) and solved the ice-age sea-level equation (*22*) using a gravitationally self-consistent pseudo-spectral algorithm (*23*). With the post-glacial sea-level changes and ice history in hand, we computed topographic change (*24*). Our calculations include shoreline migration and the feedback into sea level associated with GIA-induced perturbations in Earth rotation (*23*).

Peak modeled vertical displacement due to forebulge uplift and migration in the upper Mississippi valley occurs ~12.5 to 8.0 thousand years before present (ka) in ICE-6G/VM5a (movie S1). This is long after the LGM, which ended ~21 ka in ICE-6G (*11*), but coincides with the retreat of the main body of the LIS across the Canadian Shield (*25*). The maximum modeled uplift during ice-sheet retreat, when measured between the forebulge crest and the next GIA-induced trough away from the ice margin, whose GIA-induced deflection should only be 4.3% of that of the forebulge (*26*), reached 55 m at 10 ka B.P. (fig. S4). This is ~30 m greater than the bulge height near flexural isostatic equilibrium at the LGM and results from rapid uplift during forebulge migration. The geomorphic effect of forebulge uplift and migration is particularly pronounced in low-relief landscapes (*9*) such as the upper Midwest, whose large-river slopes (ca. 10^−4^) are similar to those associated with the glacial forebulge (ca. 2 × 10^−5^ to 2 × 10^−4^ based on our model outputs).

### Controls on the timing of bedrock incision

Rivers incise into bedrock through both abrasion and quarrying of the channel bed (*27*), neither of which can occur when the incoming flow carries and deposits enough sediment to deeply bury the bedrock valley floor, thus shielding it from erosion (*28*). These high sediment loads and the associated aggradation are common in proglacial outwash river systems: At the LGM, LIS-sourced sediment caused up to 35 m of aggradation above the modern bed of the Mississippi and its tributaries (*29*, *30*), and this thick alluvial cover was not fully re-incised even after major, sediment-poor floods from glacial lakes Agassiz and Superior (Duluth) (*30*, *31*). Therefore, we hypothesize that bedrock incision in the Mississippi River must occur during a time in which a high and/or long-lived discharge of sediment-poor water is able to erode through overlying sediments and incise several tens of meters into the underlying bedrock.

The conditions for deep incision of the upper Mississippi River would likely be satisfied at only one point in the Pleistocene: when the Mississippi River integrated sometime between 0.8 and 2.5 Ma (see Materials and Methods) (*15*, *16*, *18*, *30*, *32*). At this point, the LIS would have advanced across northward- and eastward-flowing preglacial drainages, damming them to generate lakes (*1*). These LIS-dammed lakes rose until they overtopped their southern paleodivides, sending their outflow—including ice-sheet melt (*24*)—toward the Mississippi. This would provide a consistent and high-discharge input of sediment-poor water, enabling bedrock incision (*27*, *28*, *30*) and leaving a permanent record of Mississippi River integration in the long-profile shape of the river.

**Fig. 4:**
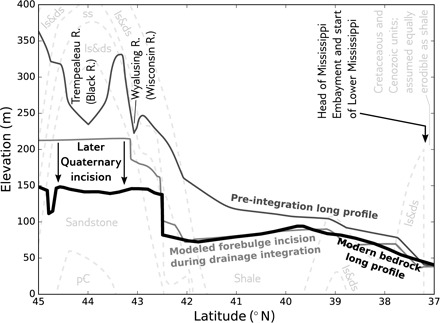
Modeled long-profile evolution. Simulated Pleistocene evolution of the long profile of the upper Mississippi River. Light gray dashed lines indicate lithologic boundaries; shale is the most erodible and limestone and dolostone are the least erodible of those rock units encountered by the river. The pre-integration long profile was modified from our Pliocene surface to account for ~65 m of Pleistocene incision, calibrated to match the elevations of the Bridgeport Terrace in the Wisconsin River Valley (*16*) and the bedrock profile of the upper Mississippi south of St. Louis (Fig. 3). The incision model used a stream-power law with variable lithology and was forced by a GIA simulation based on the algorithm of (*23*) and the ICE-6G/VM5a ice-Earth model pairing (*11*) during the drainage reversal. pЄ, Precambrian (Midcontinent Rift) basement; ss, sandstone; ls&ds, limestone and dolostone.

The depth of scour in the upper Mississippi bedrock long profile is consistent with transient uplift during forebulge migration and ice retreat (Fig. 4 and fig. S3). This caused greater vertical deflections and associated erosion than can be achieved by a stationary ice load (*26*). Therefore, the divide-overtopping flooding that began at the time of maximum ice advance continued during early stages of LIS retreat. The additional meltwater would have intensified this flooding and its erosion through the shales and sandstones of the Illinois Basin (Fig. 4), in a way that is reminiscent of divide-overtopping floods since the LGM (*13*).

Our argument for a single incision event during initial integration of the Mississippi River is bolstered by geomorphic evidence for rapid incision, which is unusual in a cratonal landscape. Incised and often bedrock-cored meanders are common on upper Mississippi River tributaries (*33*). These indicate rapid (⪆1 mm year^−1^) incision in response to sudden base-level fall, such as that which may follow drainage reversal (*1*).

### Lithology

In addition to glacially mediated drainage reorganization, forebulge uplift, and meltwater input, we hypothesize that the varied geology of the upper Mississippi valley exerts a major control on the shape of the bedrock long profile. At least 15 m of incision are unaccounted for by response to forebulge uplift, but may be associated with a weaker lithology into which a lower-slope stream can incise. To test this hypothesis, we produced a geologic cross section that follows the pre-LGM course of the Mississippi River and generalized it into zones with sedimentary rock of similar erodibilities (data files S2 and S3 and fig. S2). In order of increasing erodibility, the main geologic units are carbonates (limestones and dolostones), sandstones, and shales.

### Mississippi River incision

Once erosive, sediment-poor flows entered the paleo-Mississippi, they interacted with both the glacial forebulge and the underlying lithology. Visual inspection of our mapped bedrock long profile (Figs. 3 and 4) indicates that (i) there is an overdeepening whose shape is consistent with that of a partial glacial forebulge cast (Fig. 1) and (ii) that this overdeepening was incised almost exclusively across shales and the erodible St. Peter Sandstone, with its sharp northern knickpoint at 42.5°N stalled against the more competent carbonate bedrock of the Paleozoic Plateau (Fig. 4 and figs. S2 and S4). This overdeepening is revealed by several depth-to-bedrock measurements, but the majority of the depth-to-bedrock measurements align with the monotonically downstream-sloping bedrock long profile south of 39.7°N (Fig. 3). This pattern indicates that the overdeepening (i) forms a bedrock inner gorge that incised but did not have time to widen and (ii) was scoured below a long profile that grades into the Mississippi Embayment, which marks the end of the upper Mississippi River and sets its downstream elevation boundary condition (Fig. 2B). The inner-gorge morphology of the overdeepening, as well as its existence within the unglaciated–to–infrequently glaciated Paleozoic Plateau region (*1*), supports a fluvial rather than glacial origin.

To test whether, and under what conditions, the three aforementioned factors—sudden drainage integration, forebulge uplift, and varied lithology—can explain the long profile of the Mississippi, we integrated them into a model of river channel long-profile evolution (see Materials and Methods and the Supplementary Materials). We started with the reconstructed Pliocene surface and forced it to evolve into a pre-integration Pleistocene surface through coupled stream power–based fluvial erosion (*21*) and hillslope linear diffusion with ~65 m of early Quaternary base-level fall. At this point, the evolved pre-integration long profile matched the elevations of both the Bridgeport strath terrace in the Wisconsin River valley (*1*, *16*, *30*) and the downstream end of our reconstructed bedrock long profile (Figs. 3 and 4). This pre-drainage integration incision is critical to preserve evidence of a forebulge: If forebulge incision occurred first, then the subsequent ~65 m of base-level fall–induced incision would erase its signature. It also provides a time marker because a base-level fall signal must have propagated up the pre-integration Mississippi before incision took place. Base-level fall of ~60 m below present-day sea level was only achieved 0.5 Ma after the end of the mid-Pliocene Warm Period, that is, after 2.5 Ma B.P. (*34*), which is also the geologically constrained maximum age of incision (*15*, *17*). We next added 3 × 10^5^ m^3^ s^−1^ of annual meltwater to the upstream end of our model domain (45°N); this value is based on the annual melt-season flood under LGM discharge conditions (*24*, *35*). Last, we prescribed as an initial condition the incision of the gorge that formed immediately following the divide-overtopping flood and allowed 20,000 years of long-profile evolution based on the half-period of climate oscillations before the mid-Pleistocene transition (*36*).

Our model results (Fig. 4) reproduce the overdeepening across the Illinois Basin and the steep knickpoint near Dubuque by including a 25× higher erodibility (*K*_sp,Q_ in Eq. 1) for shales, and a 20× higher erodibility in the nearly unconsolidated St. Peter Sandstone, than in carbonate bedrock (see Supplementary Materials and Methods) (*27*, *37*). Furthermore, when we remove any component from our model (enhanced discharge, forebulge uplift, or variable lithology), the results fail to reproduce the shape of the incised forebulge (fig. S5). Our model also produces a flat-to-reverse–sloping surface to the north of the buried knickpoint (Figs. 3 and 4). While we do not explicitly model incision after the drainage integration event that produced the long-profile overdeepening, this observed flat surface may result from multiple cycles of forebulge uplift and fluvial bevelling over the remainder of the Quaternary, until the progressive incision was deep enough to near-permanently bury the bedrock surface beneath alluvium.

## DISCUSSION

Finding the buried LIS forebulge in the upper Mississippi valley demonstrates that low-gradient rivers record subtle deformations of Earth’s surface, as hypothesized in 1963 by Frye (*14*). Its shape is similar to the modern modeled profile of GIA (*10*, *11*, *23*), and we argue that this incision should have occurred during initial integration of Mississippi River drainage, sometime between 0.8 and 2.5 Ma (*15*–*18*, *30*). This suggests that the three-dimensional shape of the early Pleistocene southern LIS was similar to that of the LGM, indicating that its presumed thin and flat shape (*36*) was either not constant through time or not uniform in space. However, there are no direct dates on the incision of the Mississippi River, and such data will be required to use the bedrock long profile of the Mississippi River as an indicator of paleo–ice-sheet shape that can help to track the evolution of the LIS across the dramatic change from 40,000- to 100,000-year glacial cycles that occurred during the mid-Pleistocene transition.

The Mississippi River incised across the LIS forebulge, and we predict that past and ongoing river–GIA interactions may be widespread across former ice margins. GIA may also deflect river courses laterally (*7*–*9*), and we propose that whether a river incises into or is deflected by GIA-induced surface uplift depends on whether its incision can keep pace with rock uplift rates, in a way that is analogous to river interactions with actively growing folds (*38*, *39*). Where drainage reversals occur, we may expect to see a sediment-filled cast of the now-subsided forebulge due to the erosive power of sediment-poor meltwater from lakes that overtop their paleodivides. Where and when drainage reversals have not occurred, we may expect instead to see a signature of the forebulge in elevations of aggradational fluvial terraces. GPS data (*6*, *40*) indicate ongoing GIA around the world, highlighting where geomorphologists may find climate-“tectonic” interactions recorded even in stable cratons.

## MATERIALS AND METHODS

We reconstructed three Mississippi River long profiles: the pre-glacial profile, the buried bedrock surface, and the modern alluvial surface. The preglacial surface was reconstructed using depth-to-bedrock datasets from state geological surveys, identifying buried pre-Quaternary strath terraces, and combining these with paleodrainage pathways (*1*, *13*) to build paleotopography using a hydrologically correct spline interpolation (*20*). To map the bedrock long profile, we collected and compiled subsurface data from the Mississippi river and its former courses. Data were sourced primarily from direct measurements (wells and geotechnical borings), and were supplemented by passive seismic measurements. Table S1 includes all of our depth-to-bedrock data points. We have generalized the locations of all water wells due to security and privacy concerns. The modern alluvial surface was reconstructed from DEMs using a smoothing and channel-carving filter to remove the stair-step effect of the locks and dams.

Bedrock geology along the long profile was digitized from geologic maps and well-drilling records. We then lumped the units by lithology, including sandstone, carbonate, and shale, along the pre-LGM course of the Mississippi. This lithologic classification was the basis for defining rock erodibility in the long-profile evolution model.

River incision calculations were based on a form of the stream-power model (*21*) that can incorporate explicit discharge variability, bidirectional flow, time-variable and spatially variable uplift and subsidence, 2D nonuniformity in rock erodibility, and hillslope diffusion across interfluves,

$$\frac{\partial z}{\partial t}=-K_{\text{sp},Q}\frac{\left|Q\right|}{b}|\frac{\partial z}{\partial x}|+K_{\text{hs}}\frac{\partial^{2}z}{\partial x^{2}}+U.$$ (1)

Here, *z* is elevation, *t* is time, *x* is downstream distance, *K*_sp,Q_ is stream-power–based erodibility by fluvial processes (lumped with flood intermittency), *Q* is the discharge of the geomorphically effective flood, *b* is channel width, *K*_hs_ is hillslope diffusivity, and *U* is uplift rate. On the basis of multiple lines of evidence (see the Supplementary Materials), we estimate 300,000 m^3^ s^−1^ of excess discharge to the Upper Mississippi to occur during each snowmelt season (3 months per year) while the drainage divide is being overtopped. We assume that no geomorphically effective discharge occurs outside of the melt season.

## Supplementary Material

http://advances.sciencemag.org/cgi/content/full/5/1/eaav2366/DC1

## SUPPLEMENTARY MATERIALS

Supplementary material for this article is available at http://advances.sciencemag.org/cgi/content/full/5/1/eaav2366/DC1 Supplementary Materials and Methods Fig. S1. GPS. Fig. S2. Bedrock cross section along the Princeton-Illinois course of the upper Mississippi River. Fig. S3. GIA-induced deflection during maximum forebulge uplift. Fig. S4. Bedrock geologic map. Fig. S5. Model tests with two components. Data file S1. Bedrock topography data. Data file S2. Bedrock geology. Data file S3. Generalized bedrock geology. Movie S1. Glacial-isostatic adjustment from the Mississippi to the Gulf of Mexico. References (*41*–*101*)
