# Supplementary material for: The Mississippi River records glacial-isostatic deformation of North America
Source: Sci Adv. 2019 Jan 30;5(1):eaav2366. doi: 10.1126/sciadv.aav2366 (PMC6353627; doi:10.1126/sciadv.aav2366)
Supplement: http://advances.sciencemag.org/cgi/content/full/5/1/eaav2366/DC1 [file supp_5_1_eaav2366__index.html]

Science Advances | Science Advances

## Supplementary Materials

**The PDF file includes:**

- Supplementary Materials and Methods
- Fig. S1. GPS.
- Fig. S2. Bedrock cross section along the Princeton-Illinois course of the upper Mississippi River.
- Fig. S3. GIA-induced deflection during maximum forebulge uplift.
- Fig. S4. Bedrock geologic map.
- Fig. S5. Model tests with two components.
- Legends for data files S1 to S3
- Legend for movie S1
- References (*41*–*101*)

Download PDF

**Other Supplementary Material for this manuscript includes the following:**

- Data file S1 (Microsoft Excel format). Bedrock topography data.
- Data file S2 (Microsoft Excel format). Bedrock geology.
- Data file S3 (Microsoft Excel format). Generalized bedrock geology.
- Movie S1 (.mp4 format). Glacial-isostatic adjustment from the Mississippi to the Gulf of Mexico.

**Files in this Data Supplement:**

- Adobe PDF - aav2366\_SM.pdf
