# Supplementary material for: The Mississippi River records glacial-isostatic deformation of North America
Source: Sci Adv. 2019 Jan 30;5(1):eaav2366. doi: 10.1126/sciadv.aav2366 (PMC6353627; doi:10.1126/sciadv.aav2366)
Supplement: http://advances.sciencemag.org/cgi/content/full/5/1/eaav2366/DC1 [file aav2366_SM.pdf]

Science Advances
AAAS

advances.sciencemag.org/cgi/content/full/5/1/eaav2366/DC1

Supplementary Materials for

The Mississippi River records glacial-isostatic deformation of North America

Andrew D. Wickert*, Robert S. Anderson, Jerry X. Mitrovica, Shawn Naylor, Eric C. Carson

*Corresponding author. Email: awickert@umn.edu

Published 30 January 2019, *Sci. Adv.* **5**, eaav2366 (2019)
DOI: 10.1126/sciadv.aav2366

The PDF file includes:

Supplementary Materials and Methods
Fig. S1. GPS.
Fig. S2. Bedrock cross section along the Princeton-Illinois course of the upper Mississippi River.
Fig. S3. GIA-induced deflection during maximum forebulge uplift.
Fig. S4. Bedrock geologic map.
Fig. S5. Model tests with two components.
Legends for data files S1 to S3
Legend for movie S1
References (*41–101*)

Other Supplementary Material for this manuscript includes the following:

(available at advances.sciencemag.org/cgi/content/full/5/1/eaav2366/DC1)

Data file S1 (Microsoft Excel format). Bedrock topography data.
Data file S2 (Microsoft Excel format). Bedrock geology.
Data file S3 (Microsoft Excel format). Generalized bedrock geology.
Movie S1 (.mp4 format). Glacial-isostatic adjustment from the Mississippi to the Gulf of Mexico.

Supplementary Materials

Supplementary Materials and Methods

Bedrock long profile

We compiled subsurface data from the Mississippi River and its former courses through the Illinois River valley and the now-buried Princeton Channel (to the east) (*12*, *14*) the Pleistocene Temporary Mississippi River channel in Iowa (*41*) (to the west), and the Dakota County valley in Minnesota (to the north), to construct the shape of its buried bedrock long profile. These data were collected from: (1) well logs and depth to bedrock reports in the published literature (*12, 42–46*); (2) passive seismic measurements made by the Minnesota Geological Survey (*19*); (3) our own passive seismic measurements, calibrated to locations where the bedrock interface was established with borehole data (*19*); (4) well logs (as scans or digitized data) from state geological surveys in Minnesota (*47*), Wisconsin (*48*), Iowa (*49*), and Illinois (*50*), as well as the Missouri Department of Natural Resources; and (5) geotechnical boring logs from the Minnesota, Wisconsin, and Missouri departments of transportation and the US Army Corps of Engineers St. Paul and Rock Island districts, available as digital or scanned borehole logs (see Table S1). The boring and well logs provide depth to bedrock where it was reached or deepest-encountered sediment where it was not.

Many of the well-log data points had to be relocated or discarded due to mislocation when Public Land Survey System (PLSS) coordinates were converted to latitude/longitude coordinates. In these cases, the center of the township and range, section, quarter-section, or quarter-quarter section were used as the well location. Because of the steep topography surrounding the Mississippi River, this made it difficult to know the starting elevation of the well, especially where the section was the most precise piece of information given. We used additional information from public records of names and addresses, street intersections, and businesses, as appropriate, to properly locate as many of these sites as possible.

We interpolated the buried long profile along these points by eye by tracing the lowest-elevation points along the long profile. While we wrote and tested automated algorithms to perform this interpolation, the sparsity and irregularity of the data led to a manual approach working best. This and all other long profiles were simply digitized according to latitude: the Mississippi flows

mostly southward, and a north–south profile allows for a more intuitive comparison with the GIA model outputs.

Modern surface long profile

We computed the upper Mississippi River course and surface (i.e., alluvial) long profile using the r.watershed algorithm (*51*) from GRASS GIS (*52*) on a SRTM-derived (*53*) digital elevation model (DEM) with pits filled. We use this as the representative modern long profile against which to compare the bedrock long profile because the pre-LGM elevation of the alluvial surface along the Princeton Channel–Illinois River course (Fig. 2a) is unknown. The modern long profile has a stair-step pattern that is the result of the locks and dams that segment the upper Mississippi River into 29 pools. We applied a smoothing and channel-carving filter to digitally remove the effect of the dams and their upstream pools. A side effect of this filter is to also remove the effect of natural riverine delta dams from the Upper Mississippi long profile; these have a minor influence on the elevation of the alluvial surface above the river over the length-scale of the entire Upper Mississippi. The remaining stair-step pattern shown in the figures is due to reaches in which the flow direction of the Mississippi deviates significantly from north–south.

Pliocene long profile

We assembled bedrock topographic data along the upper Mississippi Valley. The preglacial surface was reconstructed by compiling and rasterizing state-based bedrock surface maps (*54*–*58*), plotting preglacial river courses based on ground surface and bedrock surface morphology as well as on mapped paleo-river courses (*1*, *13*, *59*), and identifying relict surfaces (*60*). These relict surfaces include oft-buried strath terraces, plaeovalleys, and the adjacent undissected upland surfaces. We gridded this sparse set of surfaces into a continuous DEM using the spline-based interpolation method of Hutchinson (*20*), which ensures that the resultant surface is hydrologically correct (i.e., that it has integrated drainage). From this continuous DEM, we extracted bedrock elevations along a profile from the Mississippi River near the confluence with the St. Croix River, downstream of St. Paul, Minnesota, to Clinton, Iowa, whence we followed the Princeton Channel and Illinois River Valley (*13*) to the modern Illinois–Mississippi confluence, and then continued downstream to near the northern margin of the Mississippi Embayment (Fig. 2). This profile crosses paleodivides inferred from diverging dips of inferred

preglacial surfaces (*1*) and evidence for high preserved surfaces. The principal paleodivides occur along or near Military Ridge in southern Wisconsin, which is consistent with prior research (*1*, *30*, *61*).

Bedrock geology

We include bedrock geology in map view, for visualisation, and in cross-section, for visualisation and inclusion in our model of river incision and long-profile evolution. In map view – Figs. 2 and S5 – we delineate the generalized lithology based on the rock age from the Geologic Map of North America (*62*, *63*) and the correlative lithostratigraphy of mid-continental North America. We define sandstones as rocks of Cambrian age; carbonates (limestones and dolostones) as rocks of Ordovician, Silurian, Devonian, Mississippian, or Permian age; and shales as rocks of Pennsylvanian age. This classification lumps the Ordovician St. Peter Sandstone with the carbonates; we split these units in the bedrock cross-section. We make two final classifications based simply on age: Precambrian and Meso- and Cenozoic (mostly Cretaceous and younger). Precambrian bedrock does not intersect the course of the Mississippi, and we treat the Mesozoic and Cenozoic strata as identical to the shales for purposes of their erodibility.

To build the cross-section, we digitized lithostratigraphic sections from geologic maps (*44*, *64*–*72*) (Table S2) and well-drilling records (*47*–*50*) (Table S3) along the same Mississippi–Princeton–Illinois course used for the Pliocene long profile, but extended into the Mississippi Embayment. We traced individual stratigraphic units across the map by hand, and then generalized the stratigraphy into zones of Precambrian (Midcontinent Rift) bedrock, sandstones, carbonates, shales, and Cretaceous and younger bedrock. In this cross-section (and hence in the river incision model), we include the Ordovician St. Peter Sandstone as a distinct generalized lithostratigraphic unit; it is highly erodible where it crops out in the upper Mississippi valley, and the buried knickpoint at Dubuque, Iowa, eroded through the St. Peter before stalling on the carbonate bedrock. While our bedrock cross-section is coarse and generalized, no published geologic cross-section down the Mississippi River currently exists, so we include it as Fig. S2.

Timing

The date of upper Mississippi drainage integration is constrained to be Quaternary in age by three geologic markers. First, highly weathered glacial outwash deposits in north-western Illinois have a westerly provenance, from the other side of the now-entrenched upper Mississippi valley, indicating that they were deposited before the modern upper Mississippi valley formed (*15*). Our mapping of these deposits indicates that they lie on a likely strath terrace surface of the early Pleistocene upper Mississippi, southeast of Dubuque, Iowa. Second, tills of the Hersey Member of the Pierce Formation in eastern Minnesota and western Wisconsin span the modern Mississippi River valley (*73*, *74*). They therefore record the advance of an ice sheet that could have induced the drainage reversal. Furthermore, they are magnetically reversed, indicating that they were deposited prior to the Bruhnes–Matuyama transition, 0.780 Ma BP (*75*). Third, the Bridgeport Moraine in southwestern Wisconsin was deposited by ice flowing from the west, crossing the present-day Mississippi Valley (*16*). As there is no evidence of catastrophic rerouting of the Mississippi by this ice advance, and the moraine is left on the eastward-dipping pre-drainage-rerouting Bridgeport strath surface (*1*), this must have been a pre-Mississippi-integration ice advance. Neither of these ice advances is dated, but the first major advance of the LIS is dated in Missouri and Iowa to 2.4–2.5 Ma BP (*17*, *32*). Based on these constraints and oxygen isotope records (*17*, *18*, *34*), we choose 2.5 Ma BP as the upper bound constraining drainage reversal of former St. Lawrence tributaries into the Mississippi.

The Bridgeport strath terrace in Wisconsin also holds evidence for the youngest possible age for Mississippi River integration and entrenchment. Knox and Attig (*16*) sampled slackwater deposits atop the strath, which lies 30 m above the modern upper Mississippi valley floor, and found that these sediments were magnetically reversed. Therefore, the last active flow to cover the strath did so prior to the Bruhnes–Matuyama transition. The 0.8 Ma BP (Marine Isotope Stage 20) (*34*) glacial period immediately precedes the Bruhnes–Matuyama transition, so this is our younger bound for the age of drainage integration.

A buried strath surface grades linearly from ~140 m at 42°N to ~45 m at 37°N, as indicated by the linear cluster of of points in Fig. 3 that intersect bedrock. This surface is ~60 m below the modern-day alluvial river long profile, and slopes more steeply than the modern river. Therefore, it is in equilibrium with a sea level that is at least 60 m below modern. Such a lowstand first

occurred ~0.5 Myr after the end of the mid-Pliocene warm period (*34*), meaning that incision to the level of the strath must have taken place ~2.5 Ma or later. The peripheral bulge is incised below this buried strath surface, and the 65 meters of anticipated incision (Fig. 4) exceed of the maximum modeled post-LGM forebulge uplift (Fig. S3). Therefore, in order for the incised peripheral bulge to be preserved, its incision must post-date incision to the level of the strath, and therefore must occur at at or after ~2.5 Ma.

Paleo-discharge

We estimate paleo-discharge of the Mississippi River based on (1) modern discharge, (2) LGM increase in drainage area, and (3) post-LGM meltwater inputs.

We built power-law relationships between discharge, drainage area, and distance downstream using data from USGS gauging stations on the Mississippi. We then modified the downstream distance by considering ancestral headwaters near Dubuque, Iowa (42.5°N), subtracting the drainage area north of the paleodivide (~210,000 km^2), and rescaling discharge accordingly. Our empirical discharge–distance relationship is $Q = 0.0098\, x^{2.096}$, where Q is given in $m^3\ s^{-1}$ and x is given in km. This relationship is used both for discharge before the drainage integration event and the base discharge that is augmented by meltwater after drainage integration.

When the Mississippi integrated, its drainage area increased abruptly, and we compute this as the sum of two components. First, ~3235 $m^3\ s^{-1}$ of mean discharge is added from the additional drainage basin area gained between the early Pleistocene and the present, primarily in Minnesota and Wisconsin. Second, Wickert (*24*) computed the change in Mississippi River discharge due to its increased drainage basin area during full-glacial times, when the drainage divide of the Mississippi extended to the ice divide in central Canada. This corresponds to a gain of ~30,000 $m^3\ s^{-1}$ in mean annual discharge. Much of this would be released as meltwater from both snow and ice, with this drainage-area-proportional component of the total water budget being seasonally replenished by new snowfall on the ice sheet.

Third, Wickert et al. (*35*) estimated ice-sheet-sourced meltwater discharge to the Mississippi since the Last Glacial Maximum from net LIS mass loss, based on precisely-dated (*76*) oxygen stable isotope ratio (δ18O) and Mg/Ca paleothermometry measurements of Gulf of Mexico

foraminifera (G. ruber) (*77–82*). We update their estimates using new measurements of δ18O in fossil Laurentide porewater across Canada (*83*) as the ice-sheet-melt end-member in their mixing model. These data show consistent δ18O values of -20‰ to -25‰, which are significantly higher than the previously-estimated (*35*, *84*, *85*) -25‰ to -38‰. This less-extreme δ18O value requires a greater amount of water discharge to maintain the isotopic balance in the Gulf of Mexico. We also increase the modern Mississippi discharge from Wickert et al.'s (*35*) estimate, which used only the mainstem Mississippi (16,790 m^3 s^{-1}), to also include the Atchafalaya–Red River system, increasing the total discharge to the Gulf of Mexico to 18,400 m^3 s^{-1}. We then re-run their model, and obtain a characteristic meltwater discharge of 40,000 m^3 s^{-1}. When applied to the original Wickert et al. (2013) (*35*) study, these new inputs increase the expected Mississippi-routed contribution to sea-level rise during Meltwater Pulse 1A, 14,650–14,310 years BP (*86*), from 0.67 m to 1.03 m, affirming the earlier conclusion that Mississippi-routed meltwater was not a major contributor to the likely 14–18 meters of eustatic sea-level rise (*86*) during this time.

Combining all of these inputs adds ~73,000 m^3 s^{-1} in mean annual discharge. However, most of this discharge would occur during the melt season, when it was also warmer (enhancing moisture transport into the ice-covered drainage basin). We approximate that all of this water was released during a summer melt season, ~3 months long. Following this assumption, the melt-season discharge becomes ~300,000 m^3 s^{-1}, which is the value of excess geomorphically-effective discharge we apply at the headwaters of the Mississippi in our model. We consider significant geomorphic change to occur only during this part of the year.

Stream-power-based incision model

Our model for upper Mississippi long-profile evolution (Equation 1) combines stream-power-driven river incision (*21*, *87*) and linear hillslope diffusion (*88*). The use of discharge during the geomorphically-effective flood (*89*) as the first term on the right-hand side of Equation 1 differs from the more commonly-used drainage-area-based stream-power approach (*21*, *87*) by explicitly including discharge. In addition, placing absolute value bars on the discharge and slope terms, allows for flow and erosion to occur in both the positive and negative *x*-directions. These are important extensions necessary to model the effects of large changes in river discharge that are disproportionate to drainage area (i.e., ice-sheet meltwater inputs) and landscape evolution on both sides of a drainage divide. Width is defined as $b = 5Q_0^{0.5}$ based on rational regime theory (*90*, *91*), where Q_0 is discharge before drainage reversal and meltwater inputs: we consider that

this catastrophic increase in discharge produced rapid incision but only slowly affected valley width, based on our observations of a narrow buried inner gorge. The diffusive second term on the right-hand side describes erosion and evolution of the divide where hillslope processes dominate. Once the Mississippi integrates into a single river with unidirectional flow, we disable the hillslope diffusion component. Spatial variations in erodibility ($K_{sp,Q}$) are locally smoothed to maintain model stability. The model is written as a mixed implcit–explicit numerical solution to maintain flexibility while improving runtime, and is parallelized to enable a parameter search to find appropriate erodibility coefficients (K).

We discount sediment cover in the model because we presume that the sediment-free water inputs from the ice- and paleodivide-dammed lakes would rapidly remove it, and also do not directly consider the mechanism of bedrock erosion. Layered carbonates and shales likely eroded by plucking (*27*, *92*), and abrasion and direct particle detachment could be significant in both the shales and weakly-consolidated sandstones. The lack of direct glacial sediment input prevents the system from being dominated by "cover" – sediment that shields the bed (*28*) – and makes it reasonable to model the Mississippi as a bedrock river. Lacking specific information on the mechanics of Mississippi River incision, we use these first-order inferences to satisfy ourselves that our lumped-parameter modeling approach is appropriate for this system.

The fluvial erosion coefficient ($K_{sp,Q}$) values chosen are 0.002 $(m/s)^{-1}$ yr^{-1} (carbonate), 0.008 $(m/s)^{-1}$ yr^{-1} (sandstone), and 0.05 $(m/s)^{-1}$ yr^{-1} (shale). These values are modified during sediment-poor meltwater floods to 0.0021 $(m/s)^{-1}$ yr^{-1}, 0.04 $(m/s)^{-1}$ yr^{-1} (St. Peter Sandstone only), and 0.25 $(m/s)^{-1}$ yr^{-1}, respectively, to represent additional erodibility due to the tools and cover effect (*28*). The more limited increase in fluvial erodibility for the carbonate rocks represents the fact that they erode predominantly by quarrying and have a wide joint spacing (*27*, *92*, *93*). These values are not based on a calibrated parameter search, but rather are representative parameters to demonstrate the plausibility of bedrock erosion leading to long-profile evolution and knickpoint retreat during deglaciation. The relative erodibilities are consistent with results from abrasion mill experiments (*28*), and their values are similar to those from prior field and modeling studies (*94–98*) after they are rescaled from a drainage area dependence to our simple discharge dependence (Equation 1).

We do not use this stream-power-based incision model for the initial drainage-reversal-driven erosion across the paleodivide, and instead simply prescribe the existence of an initial narrow

gorge. Properly modeling lake drainage across a paleodivide requires separation of the water surface and the bed surface and possible considerations of unsteady flow; these go beyond the capabilities of a simple stream-power-based incision model. Fortunately, geologic evidence of post-LGM incision of the 110-m-deep Clearwater–Athabasca spillway by a Glacial Lake Agassiz flood suggests that initial gorge erosion can occur over the course of ~1 year (*99*). Even if this timing is an underestimate, it is so much less than the 20,000 years of modeled long profile evolution included here that we consider it to be justification for prescribing the initial paleodivide incision.

Northern overdeepening

From 45°N to 42.5°N, the bedrock surface is approximately flat. This flat surface is incised near its northern end by a paleovalley (*100*) that slopes upwards to meet the modern Mississippi River (*19*) and that we therefore interpret to be a tunnel valley produced by pressurized subglacial water. Mapping (*100*) indicates that this tunnel-valley incision must have occurred during a pre-LGM glaciation.

Supplementary Materials

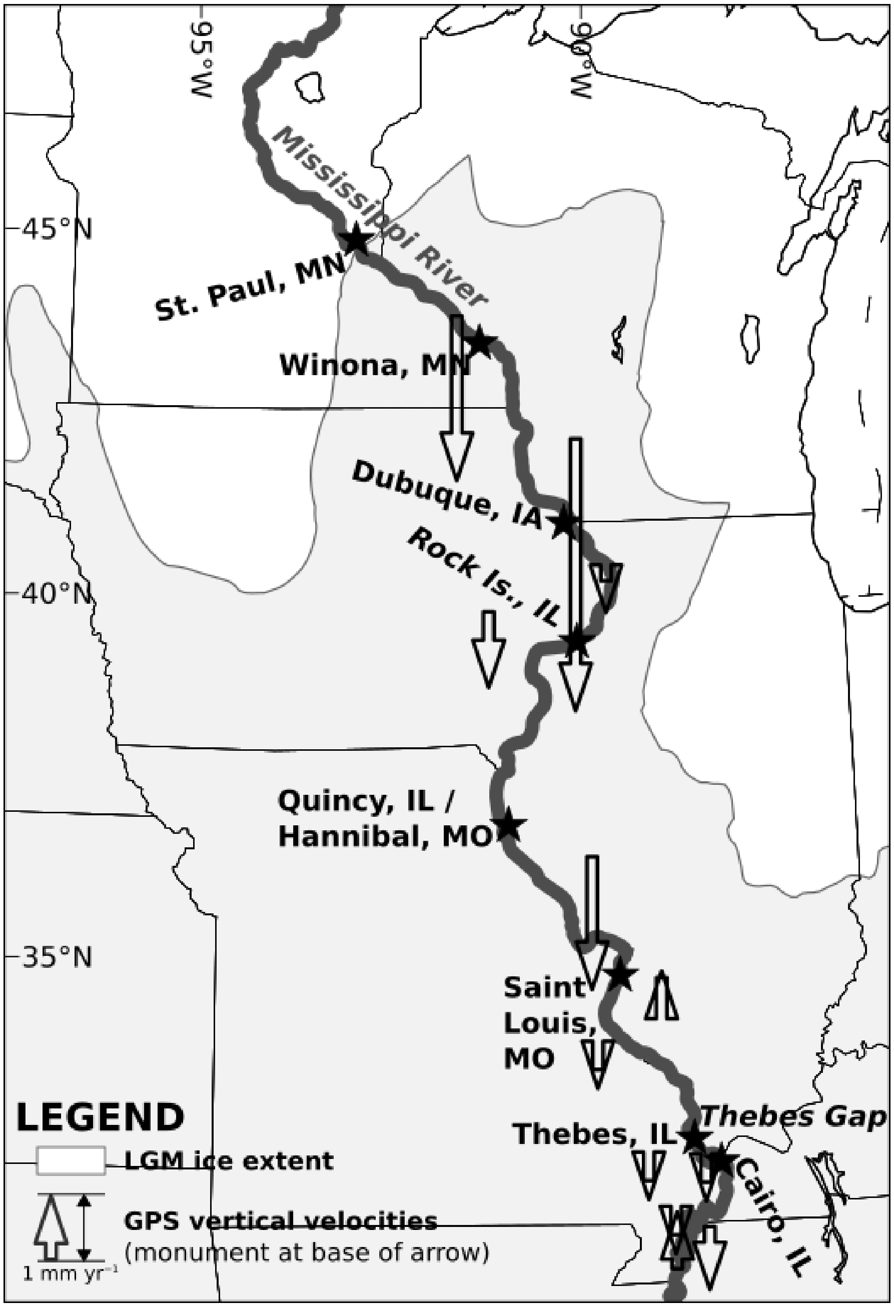

Fig. S1. GPS. GPS vertical velocities (*6*) along the Mississippi River (present-day course shown here) depict the modern collapse of the forebulge north of St. Louis, Missouri.

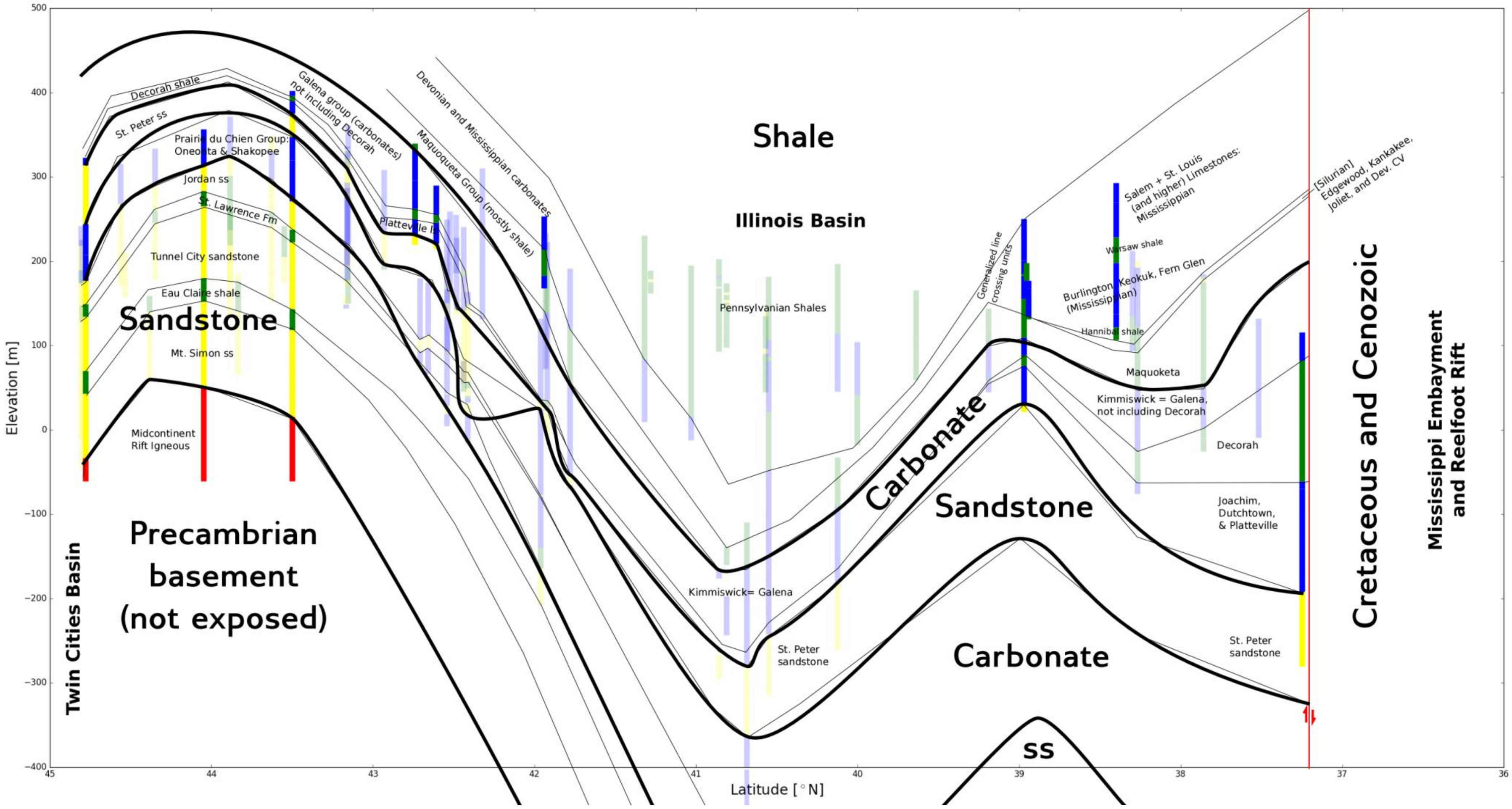

Shale
Illinois Basin
Pennsylvanian Shales
Sandstone
Carbonate
Sandstone
Carbonate
ss
Precambrian basement (not exposed)
Twin Cities Basin
Cretaceous and Cenozoic
Mississippi Embayment and Reelfoot Rift
Decorah shale
St. Peter ss
Prairie du Chien Group: Oneota & Shakopee
Jordan ss
St. Lawrence Fm
Tunnel City sandstone
Eau Claire shale
Mt. Simon ss
Midcontinent Rift Igneous
Galena group (carbonates) not including Decorah
Platteville
Maquoqueta Group (mostly shale)
Devonian and Mississippian carbonates
Kimmiswick= Galena
St. Peter sandstone
Generalized line crossing units
Salem + St. Louis (and higher) Limestones: Mississippian
Warsaw shale
Burlington, Keokuk, Fern Glen (Mississippian)
Hannibal shale
Maquoketa
Kimmiswick = Galena, not including Decorah
Decorah
Joachim, Dutchtown, & Platteville
St. Peter sandstone
[Silurian] Edgewood, Kankakee, Joliet, and Dev. CV
Elevation [m]
Latitude [° N]

Fig. S2. Bedrock cross section along the Princeton-Illinois course of the upper Mississippi River. Green is shale, blue is carbonate, yellow is sandstone, and red is Precambrian basement. Bright columns are from geologic maps; dim columns are from well logs. Generalized lithologic units used to model the bedrock long profile are separated by thick lines; thin lines delineate individual geologic units. Bedrock unit elevations used to generate the columns shown here can be found in SI Tables 2 and 3. Abbreviations – ss: sandstone; ls: limestone.

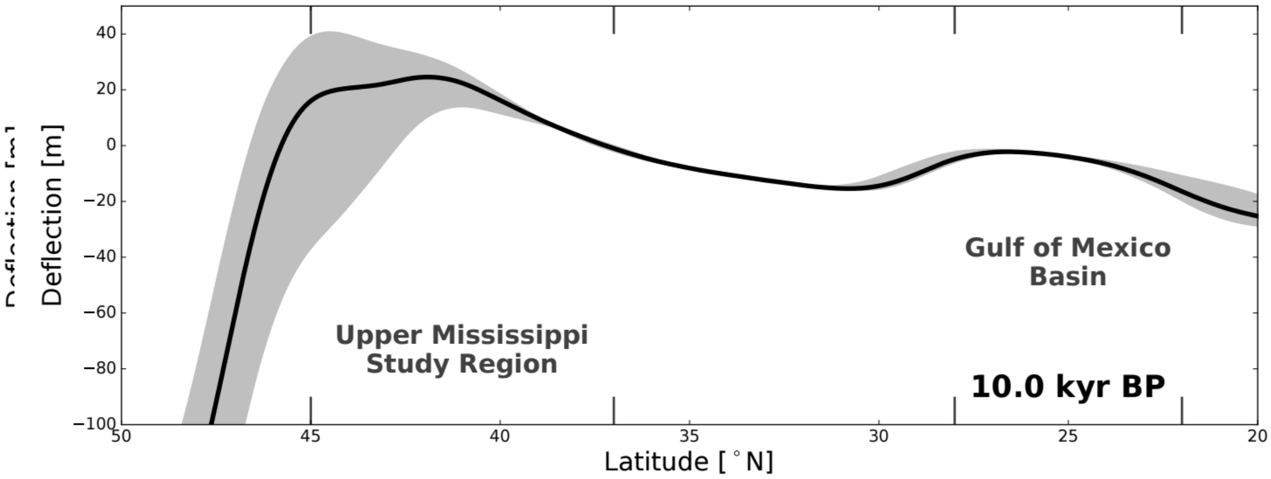

Fig. S3. GIA-induced deflection during maximum forebulge uplift. GIA-induced deflection at 10 ka BP, during maximum forebulge uplift, as modeled (*23*) using ICE-6G/VM5a (*11*). The grey field spans the range of deflections between 89 °W and 93 °W, and the black line is the mean of these deflections. The head of the Mississippi Embayment (currently Thebes Gap) at 37.2 °N is held stationary at 0 m, as this sets base-level for the upper Mississippi. Ice (un)loading since the LGM results in deep subsidence to the north and forebulge uplift in the study region. The gradually subsiding LGM uplift in the Gulf of Mexico is the result of seawater re-filling and loading the ocean basins. The entire modeled time series of GIA, from 21 ka BP to present, with 0.5 kyr time steps, may be viewed in movie S1.

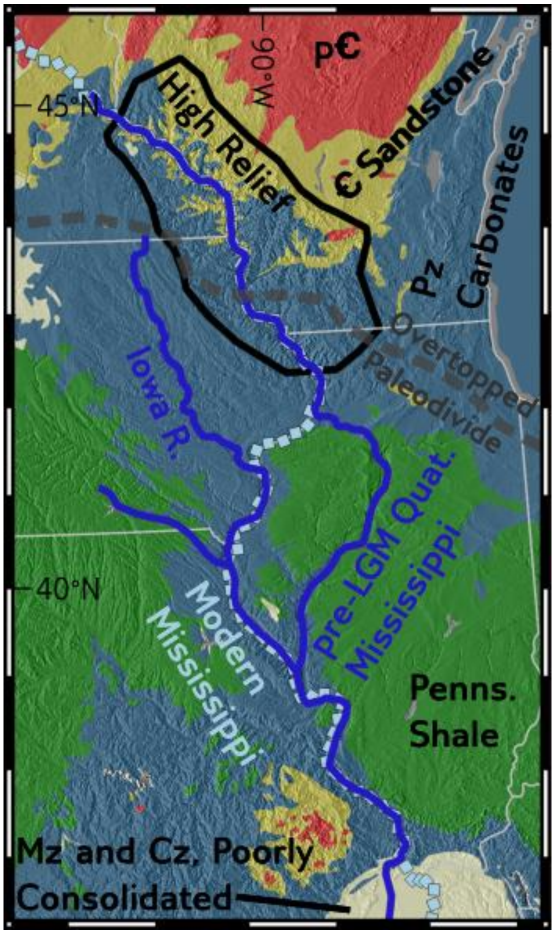

Fig. S4. Bedrock geologic map. Bedrock geologic units delineated based on age from the Geologic Map of North America (*62*, *63*) and (for the Paleozoic) the correlative Midwestern lithostratigraphy. Sandstones: Cambrian; carbonates: Ordovician through Mississippian and Permian; shales: Pennsylvanian. The easily erodible Ordovician St. Peter Sandstone is lumped with the carbonates, but contributes to deep incision at Dubuque, Iowa, where Wisconsin, Illinois, and Iowa meet. Also denoted is the high-relief area where the Paleozoic Plateau was incised following drainage reversal (*1*).

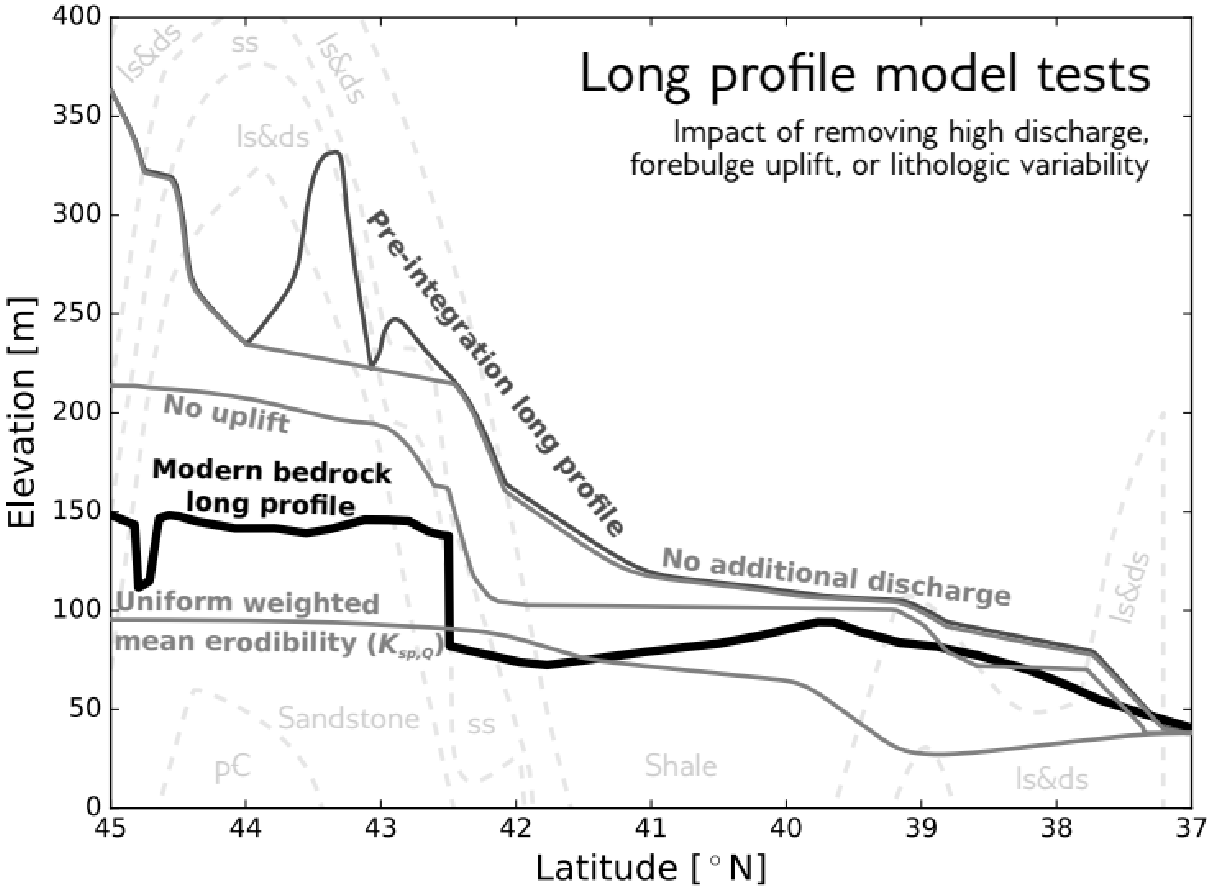

Fig. S5. Model tests with two components. Testing the model results with only two out of the three following drivers: high sediment-poor discharge, spatially-variable erodibility (assigned to lithologic units), and forebulge uplift. All three are required to produce a long-profile shape that is consistent with the buried incised forebulge and its associated knickpoint. Abbreviations – pЄ: Precambrian (Midcontinent Rift) basement; ss: sandstone; ls&ds: limestone and dolostone.

Data file S1. Bedrock topography data. Elevation of the bedrock surface along the Mississippi River and its paleochannels.

Data file S2. Bedrock geology. Bedrock geologic unit elevations along the Princeton-Illinois course of the Upper Mississippi River from geologic maps and well logs.

Data file S3. Generalized bedrock geology. Bedrock geologic unit elevations, grouped into sets with similar lithology, along the former Princeton-Illinois course of the Upper Mississippi River. These data, assembled from from geologic maps and well logs, are used to define spatially-variable bedrock erodibilities in our model.

Movie S1. Glacial-isostatic adjustment from the Mississippi to the Gulf of Mexico. Glacial-isostatic-adjustment-induced perturbations to topography from the Last Glacial Maximum to present. The grey field spans the range of deflections between 89°W and 93°W, and the black line is the mean of these deflections. The head of the Mississippi Embayment (Thebes Gap at present) at 37.2°N is held stationary at 0 m, as this sets base-level for the upper Mississippi. Ice (un)loading since the LGM results in deep subsidence to the north and forebulge uplift in the study region. The gradually subsiding LGM uplift in the Gulf of Mexico is the result of seawater re-filling and loading the ocean basins during deglacial sea-level rise.
